# An intensive multilocation temporal dataset of fungal communities in the root and rhizosphere of *Brassica napus*

**DOI:** 10.1016/j.dib.2020.105467

**Published:** 2020-04-08

**Authors:** Navid Bazghaleh, Steven D. Mamet, Jennifer K. Bell, Zayda Morales Moreira, Zelalem M. Taye, Shanay Williams, Melissa Arcand, Eric G. Lamb, Steve Shirtliffe, Sally Vail, Steven D. Siciliano, Bobbi Helgason

**Affiliations:** a University of Saskatchewan, Department of Soil Science, 51 Campus Drive, Saskatoon, SK S7N 5A8, Canada; b University of Saskatchewan, Department of Food and Bioproduct Science, 51 Campus Drive, Saskatoon, SK S7N 5A8, Canada; c University of Saskatchewan, Department of Plant Sciences, 51 Campus Drive, Saskatoon, SK S7N 5A8, Canada; d Agriculture and Agri-food Canada, 107 Science Pl, Saskatoon, SK S7N 5E2, Canada

**Keywords:** Canola, Brassica napus L, Fungal microbiome, Rhizosphere, Root

## Abstract

The plant microbiome has been recently recognized as a plant phenotype to help in the food security of the future population. However, global plant microbiome datasets are insufficient to be used effectively for breeding this new generation of crop plants. We surveyed the diversity and temporal composition of fungal communities in the root and rhizosphere of *Brassica napus*, the world's second largest oilseed crop, weekly in eight diverse lines at one site and every three weeks in sixteen lines, at three sites in 2016 and 2017 in the Canadian Prairies. 14,944 unique amplicon sequence variants (ASV) were detected based on the internal transcribed spacer region, with an average of 43 ASVs per root and 105 ASVs per rhizosphere sample. Temporal, site-to-site, and line-driven variability were key determinants of fungal community structure. This dataset is a valuable resource to systematically extract information on the belowground microbiome of diverse *B. napus* lines in different environments, at different times in the growing season, in order to adapt effective varieties for sustainable crop production systems.

Specifications Table

**Table:** 

Subject	Agriculture, Crop production, Applied microbiology
Specific subject area	Diversity and temporal composition of fungal communities in the root and rhizosphere of *Brassica napus*
Type of data	Figure
How data were acquired	DNA sequences: Illumina Miseq platform Data processing: QIIME2 platform v. 2019.1. Data analysis: R v. 3.6.1.
Data format	Raw and analyzed: (*.txt)
Parameters for data collection	Crop: sixteen diverse lines of *Brassica napus* Field sites: three sites in the Canadian prairies Materials: Root and Rhizosphere Sampling time: weekly at one site and every three weeks at three sites Years: 2016 and 2017
Description of data collection	Root and rhizosphere soil were collected and used for DNA library preparation based on amplicon sequencing of the Internal Transcribed Spacer (ITS).
Data source location	City/province (1): Llewellyn / Saskatchewan (52.1718° N, 106.5052° W) City/province (2): Melfort / Saskatchewan (52.8185° N, 104.6027° W) City/province (3): Scott / Saskatchewan (52.3574° N, 108.8400° W) Country: Canada
Data accessibility	All data are publicly available at Harvard Dataverse: An intensive multilocation temporal dataset of fungal communities in the root and rhizosphere of *Brassica napus*” Repository name: Harvard Dataverse Data identification number: https://doi.org/10.7910/DVN/DW2IUT Direct URL to data: https://dataverse.harvard.edu/dataset.xhtml?persistentId=doi:10.7910/DVN/DW2IUT Raw sequences Repository name: National Center for Biotechnology Information (NCBI) Data identification number: BioProject PRJNA575004 Accession numbers: SAMN13414364 - SAMN13415317; SAMN13416986 - SAMN13417833; SAMN13416203 - SAMN13416971

## Value of the data

• This dataset characterizes the fungal microbiome in the root endosphere and rhizosphere of *B. napus* in the Canadian prairies.
• It can be used to systematically extract information on diversity and composition of the root and rhizosphere fungal microbiome in diverse *B. napus* lines, in different environments, and at different times in the growing season.
• The data presented in this article are useful in various areas including microbial ecology, soil science, plant science, and in breeding programs as an alternative plant phenotype, in order to adapt effective varieties for sustainable crop production.

## 1 Data description

This dataset provides information on the diversity and temporal composition of the fungal microbiome in the root endosphere and rhizosphere of *Brassica napus* L. in different environments in the Canadian prairies. Previous reports revealed that root microbiomes of *B. napus*, the world's second largest oilseed crop, was consistently different from those of other crop plants, and tended to shift in different cropping rotation systems [1], [2], [3], [4]. However, no evidence was provided on the controlling factors, including how plant genetics and environment may shape the diversity and composition of this microbiome [5,6].

We surveyed the diversity and composition of the root and rhizosphere fungal microbiome of sixteen genetically diverse *B. napus* lines replicated three times. A total for four site years included sites at Llewelyn in 2016 and 2017 as well as Melfort and Scott in 2017 only. A temporally intensive survey was performed once per week for ten weeks in 2016 at Llewelyn to determine the degree of change in the fungal microbiome over the growing season. In 2017, we repeated this work at three time points with the same 16 lines grown in multiple locations (Llewelyn, Melfort and Scott). These locations had different soils and climatic factors that are representative of important canola producing regions in the Canadian prairies. In 2017, we also repeated the weekly sampling of a subset of eight lines at the Llewelyn field site (Table 1).

**Table 1:** Description of the *B. napus* lines used in this study.

*B. napus* Line	Description	Origin	Sampled at weeks 3, 6 and 9	Sampled weekly
NAM 0	Breeding Line	Canada	√	√
NAM 13	Cultivar	Germany	√	√
NAM 14	Cultivar	Sweden	√	
NAM 17	Breeding Line	Canada	√	√
NAM 23	Accession	North Korea	√	
NAM 30	Cultivar	European	√	
NAM 32	Accession	South Korea	√	√
NAM 37	Cultivar	Australia	√	√
NAM 43	Accession	Bangladesh	√	√
NAM 46	Accession	South Korea	√	
NAM 5	Accession	India	√	
NAM 72	Breeding Line	Canada	√	√
NAM 76	Cultivar	Canada	√	
NAM 79	Accession	Pakistan	√	
NAM 48	Breeding Line	Canada	√	
YN04-C1213	Breeding Line	Canada	√	√

**Fig. 1:**
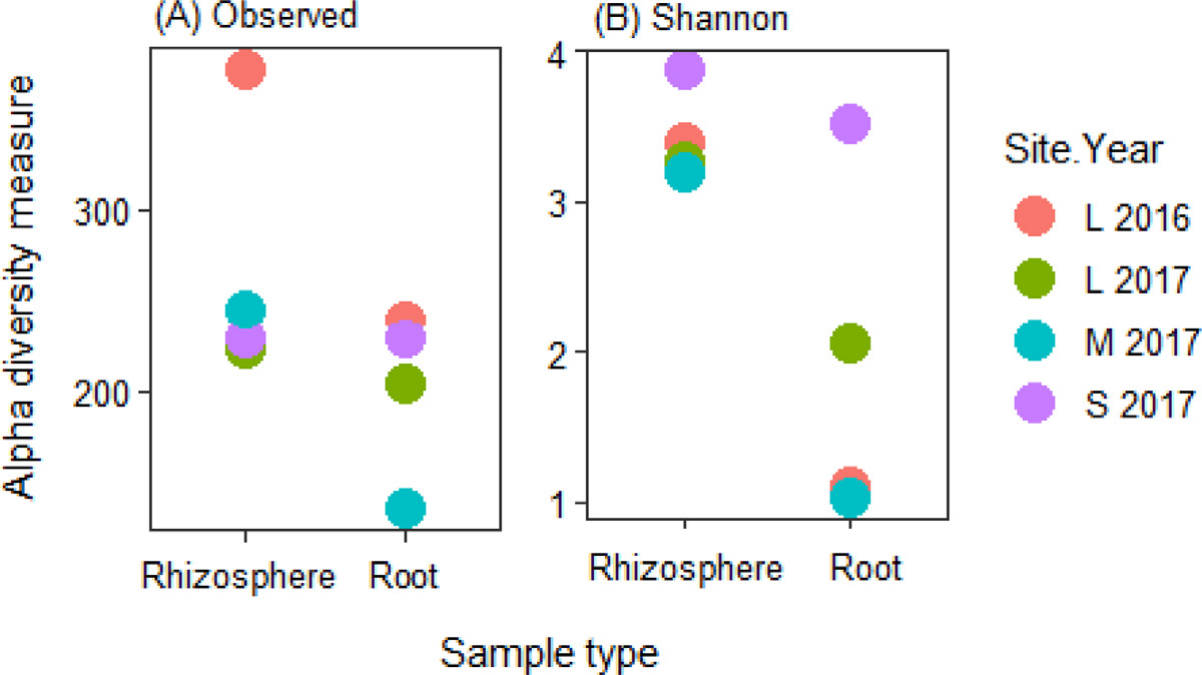
Alpha diversity in each site in a given year. (A) Observed species, and (B) Shannon's *H* index, explaining the diversity of fungal taxa. L refers to Llewellyn, M to Melfort, and S to Scott in 2016 and 2017.

**Fig. 2:**
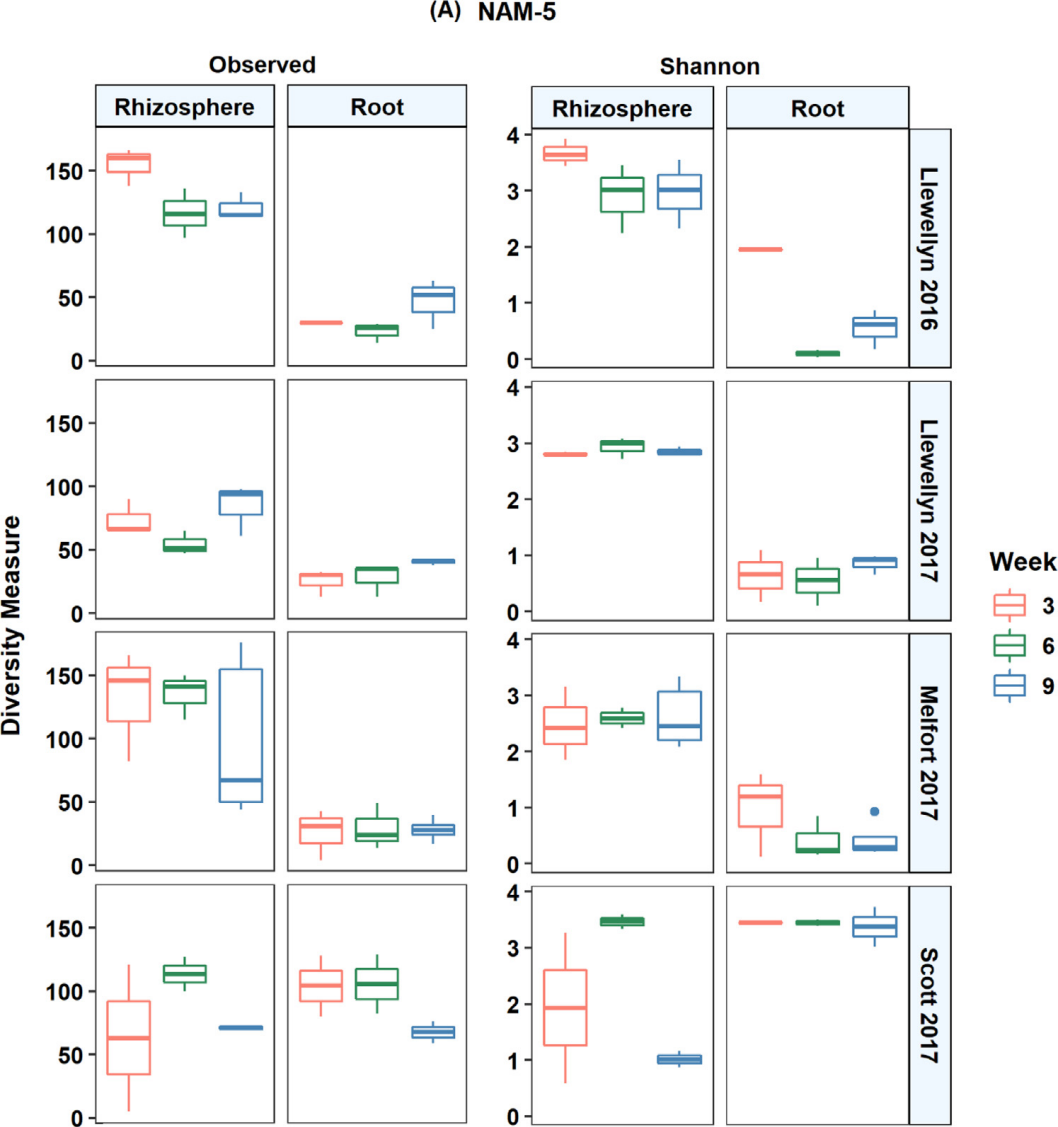

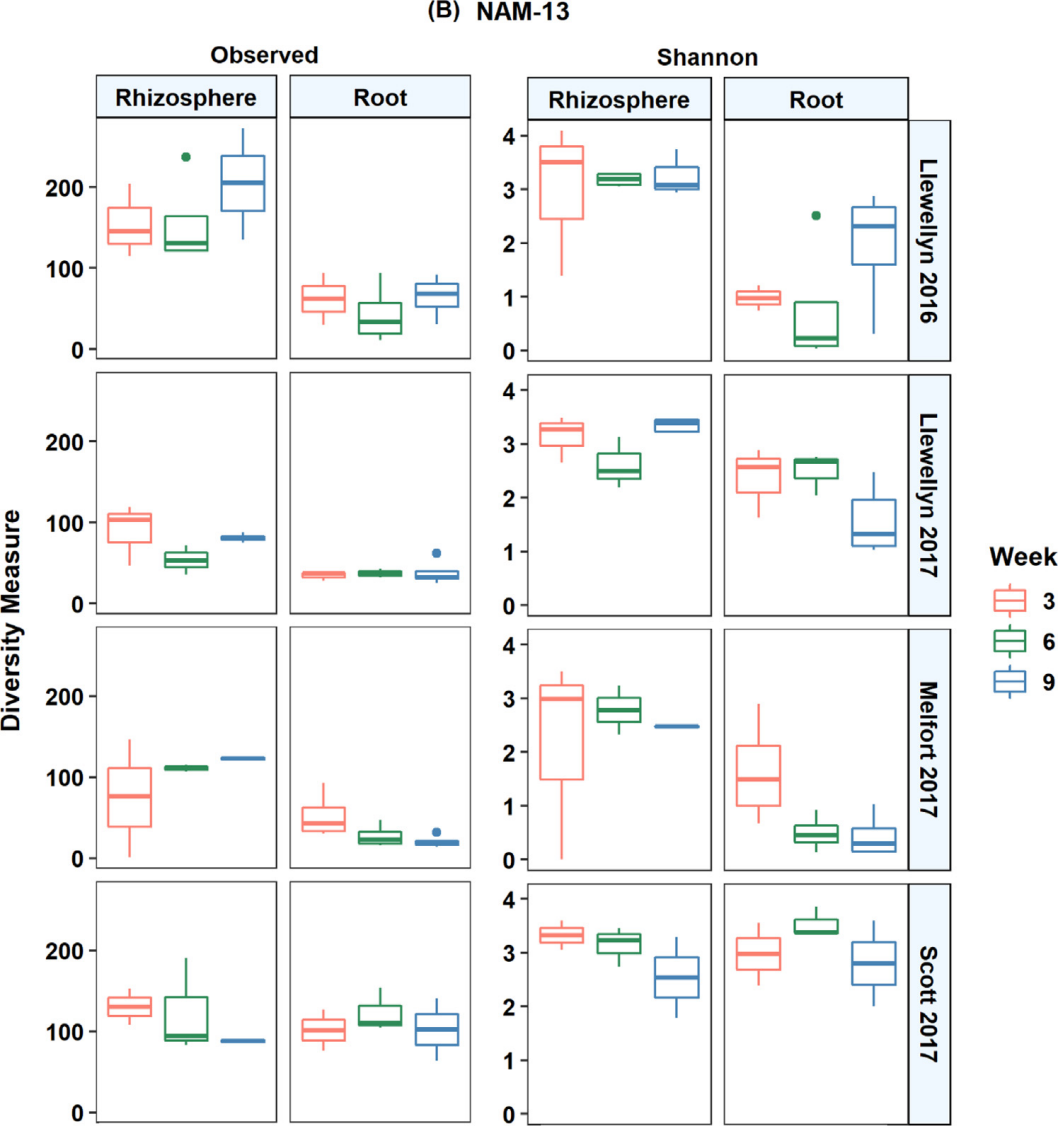
Alpha diversity in two canola lines over growing seasons in each site in a given year. Observed taxa and Shannon's *H* index of diversity of fungal taxa in the roots and rhizosphere of two canola lines including NAM-5 (A) and NAM 13 (B) at weeks 3, 6, and 9 in 2016 and 2017.

**Fig. 3:**
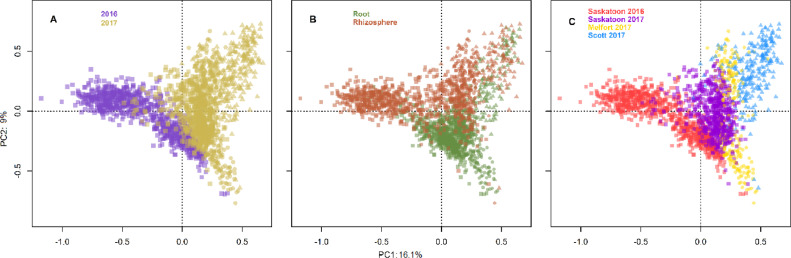
Principal components analysis of fungal taxa obtained using ITS amplicon sequencing in different years (A), sample types (B) and sites (C). Permutational Analysis of Variance (PERMANOVA) shows significant effects of year (*p* <0.001*)*, sample type (*p* <0.001*)*, and site by year (*p* <0.001*)* on fungal microbiome composition.

In total, 14,944 unique amplicon sequence variants (ASV) were detected in all lines across the four site years based on the internal transcribed spacer region. There were an average of 43 ASVs per root and 105 ASVs per rhizosphere sample. The fungal microbiome was more diverse in the rhizosphere samples compared to the root samples and it was more diverse at Scott compared to Llewellyn and Melfort. In Llewellyn, the site with two years of data, the root fungal microbiome was more diverse in 2017 than 2016, while in contrast in the rhizosphere soil the microbiome had a higher richness in 2016 than 2017 (Fig. 1). Diversity of fungi varied in different *B. napus* lines and it shifted over growing seasons. An example of this is shown for lines NAM-5 and NAM-13 (Fig. 2). The composition of fungal communities in the root and rhizosphere was shaped by *B. napus* line, location, and the year the experiment was conducted, likely dominated by year-to-year differences in environmental and edaphic conditions (Fig. 3).

This dataset is a valuable resource that can be used to systematically extract information on the temporal dynamics of fungal communities in the root endosphere and rhizosphere soil of diverse *B. napus* lines in different soils and across years. The purpose of this report is to provide a publicly available fungal dataset, its associated metadata and the context by which others explore how fungal microbiomes relate to varieties better suited to sustainable crop production, and improve food security worldwide [7], [8].

## 2 Experimental design, materials, and methods

### 2.1 Experimental design

Sixteen lines of *B. napus* L. were grown in Agriculture and Agri-Food Canada research farms in Saskatchewan in 2016 and 2017 (Table 1). In 2016, the lines were grown in a site at Llewellyn (52.1718° N, 106.5052° W). In 2017, the same lines were again grown at Llewellyn, as well as at Scott (52.3574° N, 108.8400° W) and Melfort (52.8185° N, 104.6027° W). Each line was grown in randomized blocks and replicated three times. Each plot had six rows and was 6.1 m long and 1.8 m wide. At Llewellyn in 2016, the plots were seeded on May 27 and received 220.1 mm of precipitation throughout the growing season (May to August) and had a mean temperature of 16.7 °C. In 2017, the plots were seeded on May 28–29 at Llewellyn and received 127.9 mm of precipitation throughout the growing season with a mean air temperature of 16.4 °C. At Melfort, the plots were seeded on May 19, 2017 and received 126.9 mm of precipitation throughout the growing season with a mean temperature of 15.5 °C. At the Scott site the plots were seeded on June 20, 2017 and received 178.7 mm precipitation with a mean temperature of 15.4 °C. These plots were initially seeded in May but the plants were lost due to a hailstorm and they were reseeded later in June.

In 2016, root and rhizosphere samples were collected weekly from June 14 to August 16 from the Llewellyn site for 10 consecutive weeks, starting 3 weeks after sowing. In addition, three duplicates were collected for randomly chosen samples at each sampling time throughout the growing season resulting in a total of 510 samples. In 2017, the same sixteen lines were sampled at all three sites 3, 6, and 9 weeks after sowing. In 2017, eight lines were also sampled weekly for 10 weeks from the site at Llewellyn, starting 3 weeks after sowing. Three canola plants were collected and combined to form a single composite sample from each plot. A total of 2160 root and rhizosphere samples were collected and DNA was extracted from all samples. Roots and rhizosphere samples were collected to a 10-cm depth, and rhizosphere soil was determined as soils adhering to the roots.

### 2.2 Sample collection and processing

Three plants including roots were sampled from each plot using a sterilized trowel. Roots with adhering soil and bulk soil were placed in a sampling bag and stored in a cooler where they were kept on ice. The samples were transported to the lab and were stored at 4 °C until processing. The following day, the aboveground material was cut from the roots, oven dried at 60 °C, and weighed. The loosely adhering soil (soil not attached to the roots) was shaken off and collected and stored at −80 °C for further analysis. Plant roots and the tightly adhering soil were weighed and transferred to a flask containing 100 ml of sterile 0.05 M NaCl buffer and shaken at 180 rpm for 15 min. The roots then were rinsed with deionized water, weighed, and a subsample of the root material was stored at −80 °C for DNA extraction. The buffer and soil mixture was centrifuged at 5000 rpm for 15 min at room temperature. The supernatant was stored at −20 °C for root exudate analysis. The pellet containing the rhizosphere soil was transferred to 1.5 ml tubes and stored at −80 °C for DNA extraction.

### 2.3 DNA extraction and amplification

DNA was extracted from 250 mg of rhizosphere soil using Qiagen PowerSoil extraction kit following manufacturer instructions. DNA was extracted from 50 mg root tissue using Qiagen PowerPlant extraction kit (Hilden, Germany) following manufacturer instructions. Extraction duplicates were included for quality control. After extraction, DNA quantity and quality were determined following the standard Qubit protocol (Thermo Fisher Scientific, Waltham Massachusetts). Prior to amplification, DNA from soil was standardized to 5 ng/µL, and DNA from roots was standardized to 1.5 ng/µL. The Internal Transcribed Spacer (ITS) region was amplified using the ITS1F_KYO1 (CTHGGTCATTTAGAGGAASTAA) / ITS2_KYO2 (TTYRCTRCGTTCTTCATC) primer set [9] and then sequenced using the Illumina MiSeq Platform. The PCR reaction mix (25 µL total) contained 12.5 µL Platinum Green Taq buffer (Invitrogen, Carlsbad, California), 1 µL of each of the forward and reverse primers (10 µM), 8.5 µL nuclease free water, and 2 µL of template DNA. The PCR conditions were 95 °C for 5 min as an initial denaturation, followed by 95 °C for 30 s, 51 °C for 45 s, 72 °C for 1 min for 35 cycles, and a final elongation of 72 °C for 7 min. PCR reactions with no DNA template were included for negative control. PCR reactions were replicated for a fraction of randomly selected DNA samples and used for PCR quality control.

### 2.4 Library preparation

The PCR products were confirmed by visualization in an agarose gel (1.2%), purified using 1:1 ratio of Nucleomag NGS clean-up and size selected to remove primers and impurities according to manufacturer's instructions (D-mark Biosciences, Scarborough, Ontario). After purification, samples were barcoded using Nextera XT indexes, purified again to remove the impurities and indexing primers, quantified using Qubit 4 (Thermo Fisher Scientific, Waltham Massachusetts), standardized at 4 ng/µL, and pooled (384 samples). Selected PCR reactions were indexed separately and used for sequencing quality control.

Pooled libraries were then sequenced using the Illumina MiSeq platform using MiSeq Reagent Kit v2 (500-cycles). A total of 1080 individual root samples and 1080 rhizosphere soil samples, as well as 411 duplicates were sequenced. The raw sequences were submitted to the sequence read archive (SRA) repository of the National Center for Biotechnology Information (BioProject PRJNA575004, Accessions: SAMN13414364 - SAMN13415317; SAMN13416986 - SAMN13417833; SAMN13416203 - SAMN13416971).

### 2.5 Bioinformatics

In total, 113,378,162 forward and reverse raw sequence reads were produced with an average of 26,244 paired reads per sample. Sequences were processed using QIIME2 v2019.7 [10]. First primer sequences were removed. Sequences were quality filtered using the default parameters and arranged as amplicon sequence variants (ASVs) using a forward truncation length of 180 bp and reverse truncation length of 120 bp in DADA2. There were 14,944 unique ASVs with an average of – 43 ASVs per root and 105 ASVs per rhizosphere sample. The average sequence length for both root and rhizosphere was 238 base pairs. ASVs were classified using UNITE database v 8.0 [11]. The QIIME2 abundance and taxonomy artifacts were combined and converted to BIOM format for processing in R v. 3.5.3. The fungal abundance data were imported into R using the biomformat package v. 0.4.0 and combined with the taxonomy and sample information using phyloseq v. 1.26.1 [12]. Duplicate samples, and samples or taxa with zero abundance sums were removed.

### 2.6 Statistical analysis

Zeros in the dataset were replaced using a Bayes-Laplace approach using the zCompositions v. 1.2.0 R package and then transformed using a centered-log ratio (CLR) transformation using the CoDaSeq v. 0.99.3 package [13], [14], [15]. Initial exploration of the composition of the fungal community was assessed using Principal Component Analysis (PCA) and compositional differences quantified using a Permutational Analysis of Variance (PERMANOVA) in the vegan v. 2.5–6 package in R [16]. Alpha diversity was calculated using vegan v. 2.5–6 in R.

## Declaration of Competing Interest

The authors declare no competing interests

## Appendix Supplementary materials

Image, application 1
